# Structural basis of DNA polymerase θ mediated DNA end joining

**DOI:** 10.1093/nar/gkac1201

**Published:** 2022-12-30

**Authors:** Chuxuan Li, Hanwen Zhu, Shikai Jin, Leora M Maksoud, Nikhil Jain, Ji Sun, Yang Gao

**Affiliations:** Department of Biosciences, Rice University, Houston, TX 77005, USA; Department of Structural Biology, St. Jude Children’s Research Hospital, Memphis, TN 38105, USA; Department of Biosciences, Rice University, Houston, TX 77005, USA; Center for Theoretical Biological Physics, Rice University, Houston, TX 77005, USA; Department of Biosciences, Rice University, Houston, TX 77005, USA; Department of Structural Biology, St. Jude Children’s Research Hospital, Memphis, TN 38105, USA; Department of Structural Biology, St. Jude Children’s Research Hospital, Memphis, TN 38105, USA; Department of Biosciences, Rice University, Houston, TX 77005, USA

## Abstract

DNA polymerase θ (Pol θ) plays an essential role in the microhomology-mediated end joining (MMEJ) pathway for repairing DNA double-strand breaks. However, the mechanisms by which Pol θ recognizes microhomologous DNA ends and performs low-fidelity DNA synthesis remain unclear. Here, we present cryo-electron microscope structures of the polymerase domain of *Lates calcarifer* Pol θ with long and short duplex DNA at up to 2.4 Å resolution. Interestingly, Pol θ binds to long and short DNA substrates similarly, with extensive interactions around the active site. Moreover, Pol θ shares a similar active site as high-fidelity A-family polymerases with its finger domain well-closed but differs in having hydrophilic residues surrounding the nascent base pair. Computational simulations and mutagenesis studies suggest that the unique insertion loops of Pol θ help to stabilize short DNA binding and assemble the active site for MMEJ repair. Taken together, our results illustrate the structural basis of Pol θ-mediated MMEJ.

## INTRODUCTION

DNA double-strand breaks (DSBs) are the most deleterious form of DNA damage. If not repaired properly, a single DSB is sufficient to induce genome rearrangement and cell apoptosis. In eukaryotes, there are three complementary pathways responsible for repairing DSBs: homologous recombination (HR), non-homologous end joining (NHEJ), and microhomology-mediated end joining (MMEJ) (1,2). In contrast to NHEJ that requires no homologous ends and to HR that utilizes long homologous strand for repair, MMEJ relies on very short microhomology of resected DNA ends (2-6 base pairs, bp) (3–5) at DSBs. MMEJ is highly error-prone and often leads to insertions, deletions, and mutations at the break site (6,7). It is initially thought that MMEJ serves as a backup repair mechanism for HR and NHEJ, but its operation has been reported when the other two repair pathways are proficient (8,9). DNA polymerase θ (Pol θ) plays multiple roles in MMEJ (7,10,11). Pol θ catalyzes the annealing of microhomologous DNA ends and performs template-dependent DNA extension (12–14). A recent study suggested that Pol θ has nuclease activity to remove DNA flaps generated upon synapsis formation (15). In addition to its roles in MMEJ, Pol θ has been shown to perform error-prone translesion synthesis (TLS) across various DNA lesions (16–20). Pol θ is minimally expressed in normal somatic cells but overexpressed in many cancer cells (21). Inhibition of Pol θ is lethal in HR-deficient cancer cells with BRCA1 and BRCA2 mutations (7,10), making Pol θ-mediated MMEJ a promising target for precision cancer therapy. In fact, two Pol θ inhibitors have been recently reported for the treatment of BRCA1 and BRCA2 related cancers (22,23). Studying the working mechanisms of Pol θ will allow a comprehensive understanding of its functions in maintaining genome stability and promote the development of cancer chemotherapeutics that target Pol θ.

Human Pol θ (HsPol θ), encoded by the POLQ gene, is a 2590 amino-acid long protein with two functional domains connected by a long unstructured central domain (24). A superfamily II type helicase (Pol θ-hel) is on the N-terminus of Pol θ. During MMEJ, Pol θ-hel can strip off RPA (Replication Protein A) on resected DNA ends and facilitate the annealing of single-stranded DNA (ssDNA) with microhomologies in an ATP-dependent manner (12–14). An A-family DNA polymerase (Pol θ-pol), which contains an inactivated pseudo-exonuclease domain and a polymerase domain, is located on the C-terminus of Pol θ. Distinct from its homologs in DNA replication, Pol θ-pol exhibits low fidelity and possesses TLS activities. It can catalyze DNA synthesis on MMEJ DNA substrates with 2–6 bp of microhomology (3,4) and on DNA substrates containing various types of DNA lesions, including but not limited to thymine glycol, abasic sites (AP), and cyclobutane pyrimidine dimers (CPD) (16–20). Five unique insertion loops have been found in Pol θ-pol, two of which are located in the inactivated exonuclease domain, and the other three are in the polymerase domain (25). The insertion loops in the polymerase domain have been shown to promote MMEJ and TLS (5,16). So far, structures of HsPol θ-pol have been reported with undamaged long duplex DNA (25), long duplex DNA with an AP lesion (25) or RNA/DNA hybrids (26). During revision of the present work, another crystal structure was published of HsPol θ-pol bound to undamaged duplex DNA and an inhibitor at 2.6 Å (27). However, how Pol θ-pol performs error-prone DNA synthesis against MMEJ substrates or DNA lesions remains unclear. Moreover, the insertion loops are yet either disordered or truncated in the reported structures; how these loops contribute to the activities of Pol θ in TLS and MMEJ (5,16) requires further investigation.

In this work, we characterized a Pol θ-pol homolog from vertebrate *Lates calcarifer* (Lc), which shares high sequence and functional similarities with HsPol θ-pol. Cryo-electron microscope (cryo-EM) studies of LcPol θ-pol with long and short DNA substrates resolved three structures of LcPol θ-pol, with 18, 11, and 4-bp duplex DNA visible at overall resolutions of 2.8, 2.4 and 3.0 Å, respectively. The structures explain the mechanistic basis by which Pol θ-pol interacts with DNA and the incoming nucleotide at atomic resolution. Together with biochemical studies and computational simulations, our data elucidate unique features of Pol θ-pol that are essential for the low-fidelity MMEJ synthesis and the critical roles of the insertion loops in stabilizing short MMEJ DNA substrates and assembling the active site.

## MATERIALS AND METHODS

### Cloning, expression, and purification of wild-type and mutant LcPol θ-pol

The gene encoding the polymerase domain of LcPol θ (amino acid residues 1950–2714) was synthesized by Gene Universal and was inserted into the pETsumo2 vector containing a His_6_ and a SUMO tag. All mutants were constructed by PCR-based mutagenesis. LcPol θ-pol and mutants were expressed in Rosetta2 (DE3) cells by IPTG induction at 16°C for 18 h and were purified to homogeneity as follows. WT and mutant His_6_-SUMO tagged proteins were first cleaned by nickel gravity flow. After removal of the His_6_-SUMO tag by SENP2 protease, they were further purified through a HiTrap heparin HP column. All purification steps were performed at 4°C. Purified proteins were concentrated to 6–16 mg/ml, aliquoted and stored at –80°C in 25 mM Tris [pH 8.0], 300 mM NaCl, 3 mM dithiothreitol (DTT) and 30% glycerol. A fresh aliquot was used each time for cryo-EM sample preparation and biochemical assays.

### DNA synthesis assay

**Figure 1.**
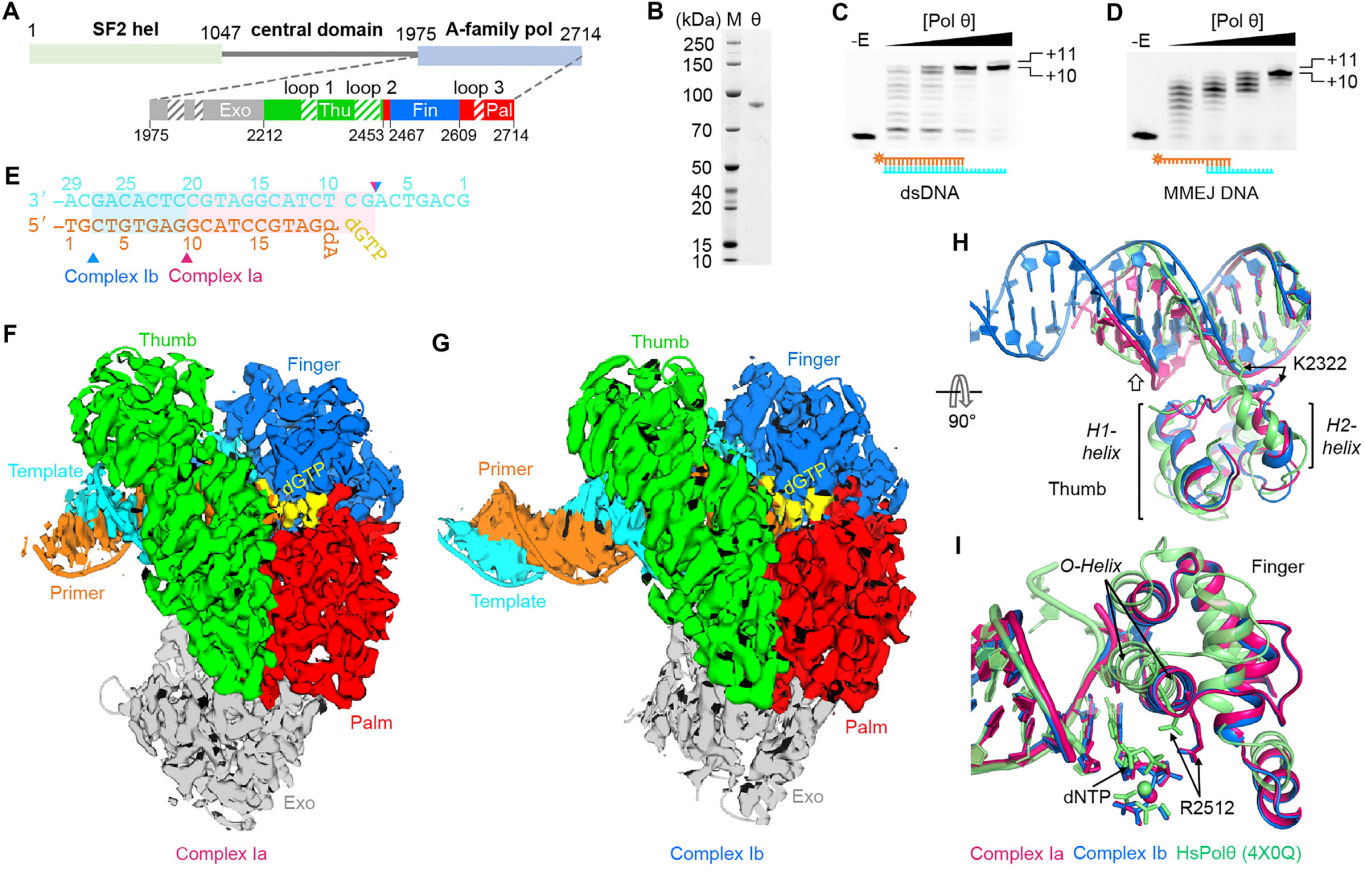
Biochemical and structural characterization of LcPol θ. (**A**) Domain structure of full-length and the polymerase construct of LcPol θ. Hel, helicase; Pol, polymerase; Exo, exonuclease; Thu, thumb; Fin, finger; Pal, palm. The exonuclease, thumb, finger and palm domains are colored grey, green, blue and red, respectively. Insertion loops are indicated by the shaded boxes. (**B**) An SDS-PAGE gel indicating the purity of LcPol θ-pol. (**C, D**) DNA synthesis activity of LcPol θ-pol on a regular primer/template dsDNA (**C**), and on a short MMEJ substrate with 6 bp of microhomology (**D**). The control samples without enzymes are shown in the -E lanes. (**E**) DsDNA substrate used for cryo-EM study. The primer and template are numbered from their 5′-ends. The transparent boxes and triangles represent visible portions of the DNA substrates in complexes Ia and Ib. (**F, G**) Overall cryo-EM structures of complex Ia at 2.4 Å (**F**) and of complex Ib at 2.8 Å (**G**). Individual domains are color-coded and the cryo-EM density map is overlaid upon the structural model. (**H, I**) Movements of the thumb and finger domains. Complex Ia (pink), complex Ib (blue) and HsPol θ (green) are superimposed with their thumb (**H**) and finger domains (**I**). The H1- and H2-helices are named following the structures of Taq polymerase (42). The incoming nucleotide (dNTP) and key protein residues are indicated by arrows.

All DNA strands used in the present work were purchased from Integrated DNA Technologies and were summarized in Supplementary Table S2. The hairpin substrates and the primer strands of the 19/29 mer and of MH6 for DNA synthesis assay have a 6-FAM label at the 5′ end. DNA annealing was carried out by incubation at 95°C for 5 min followed by cooling to 25°C at a rate of 1°C/min for the 19/29 mer and by immediate cooling on ice for the hairpin substrates. As regards MH6, which only contains 6 bp, the primer and the template strands were simply mixed at equal molar ratio before incubating with LcPol θ-pol. DNA synthesis reactions shown in Figure 1C and D took place in 10 μl of reaction buffer containing 25 mM Tris [pH 7.6], 100 mM KCl, 3 mM DTT, 100 μg/ml BSA, 5% glycerol, 100 μM dNTP for the 19/29 mer or 100 μM dATP for MH6, and 5 mM MgCl_2_. 100 nM of the 19/29 mer or MH6 and varying concentrations of WT LcPol θ-pol (5, 10, 20, 50 nM) were pre-incubated as a mixture at 25°C for 2 min and were combined with MgCl_2_ and dNTP or dATP for reaction initiation. The reactions were allowed to proceed at 25°C for 10 min before quenching by the addition of 10 μl of stop solution containing 100 mM EDTA, 80% formamide and 0.01% bromophenol blue. Reactions shown in Figure 3B and D were performed similarly except that 1, 5, 10 and 50 nM of WT LcPol θ-pol were used. Reactions shown in Figure 5C, G and H were carried out under similar conditions as well but with following alterations. 1 μM of HP [3,9] and varying concentrations of WT or mutant LcPol θ-pol (0.5, 1, 2, 5, 10, 50, 100, 200, 500 nM) were pre-incubated as a mixture at 37°C for 2 min. The reactions were initiated by the addition of MgCl_2_ and dATP and proceeded at 37°C for 5 min. To check the fidelity of WT LcPol θ-pol in template-dependent DNA synthesis, as shown in Figure 3C, reactions were carried out using 100 nM of HP [3,3] and 50 nM of the enzyme. All other parameters were the same as for the DNA synthesis assays.

**Figure 2.**
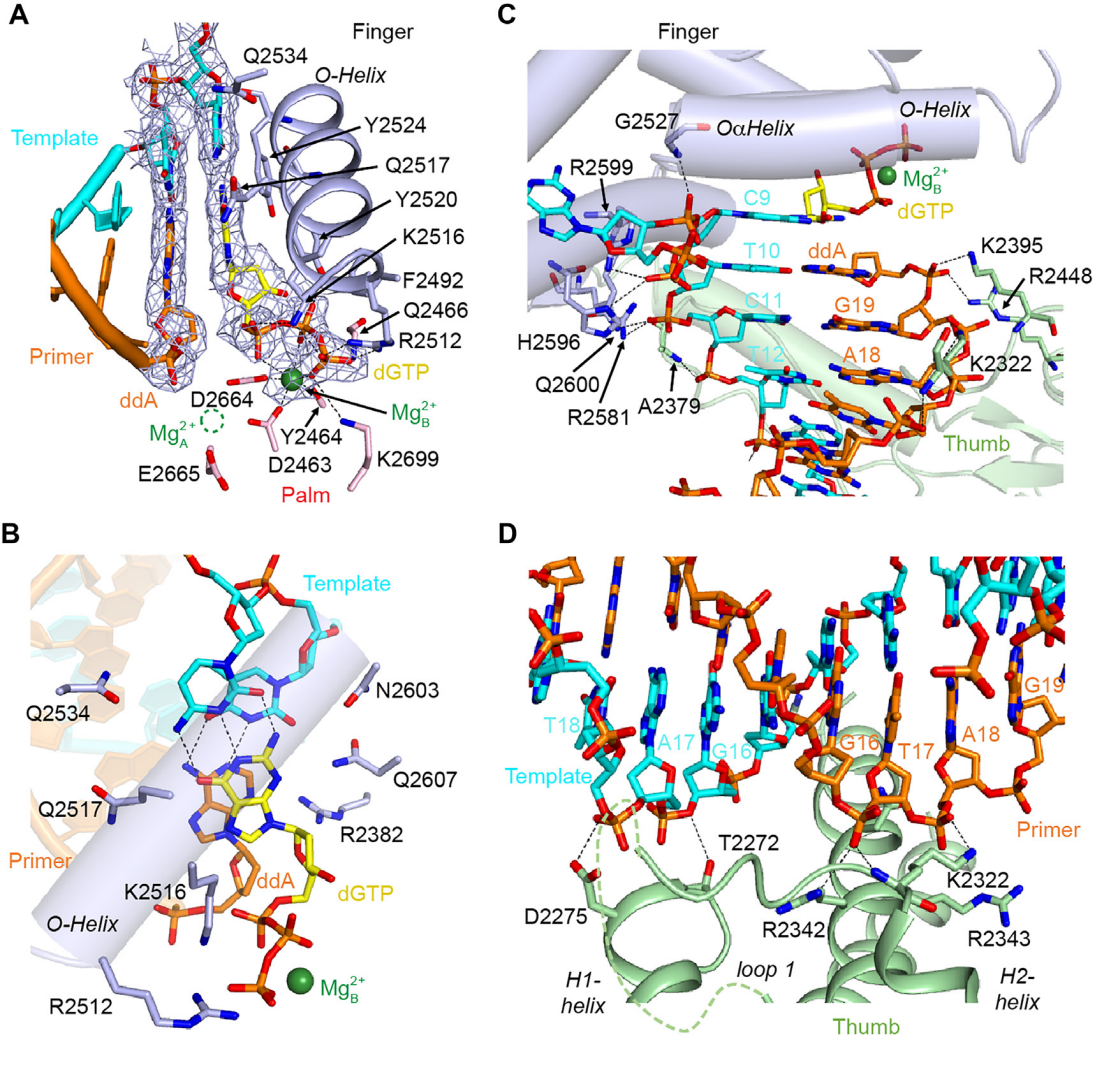
Active site and DNA interactions of LcPol θ-pol. (**A**) Active site of LcPol θ-pol. The cryo-EM map densities for the nascent and the first base pairs at the primer end are shown in meshes. (**B**) Top-down view of the nascent base pair. Hydrophilic protein side chains are labeled. (**C**) Interactions between LcPol θ-pol and the dsDNA near the active site. (**D**) Interactions between the thumb domain and the dsDNA. Key residues engaged in incoming nucleotide and Mg^2+^ binding and DNA interactions are shown in sticks and labeled.

**Figure 3.**
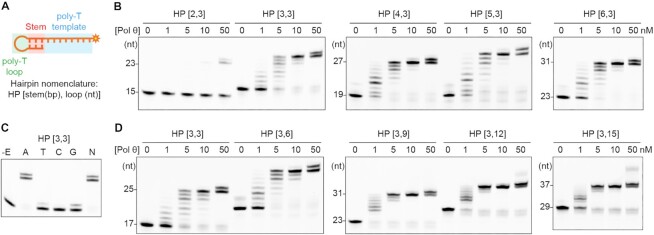
DNA synthesis assay of LcPol θ-pol on hairpin substrates. (**A**) Design of the hairpin DNA substrates. (**B**) DNA synthesis assay of LcPol θ-pol on hairpin substrates that vary in the number of base pairs in the stem region. (**C**) HP [3,3] extension by LcPol θ-pol in the presence of individual (A, T, C or G) or all four dNTPs (N). The control sample without enzyme addition is shown in the –E lane. (**D**) DNA synthesis assay of LcPol θ-pol on hairpin substrates that vary in the loop length.

**Figure 4.**
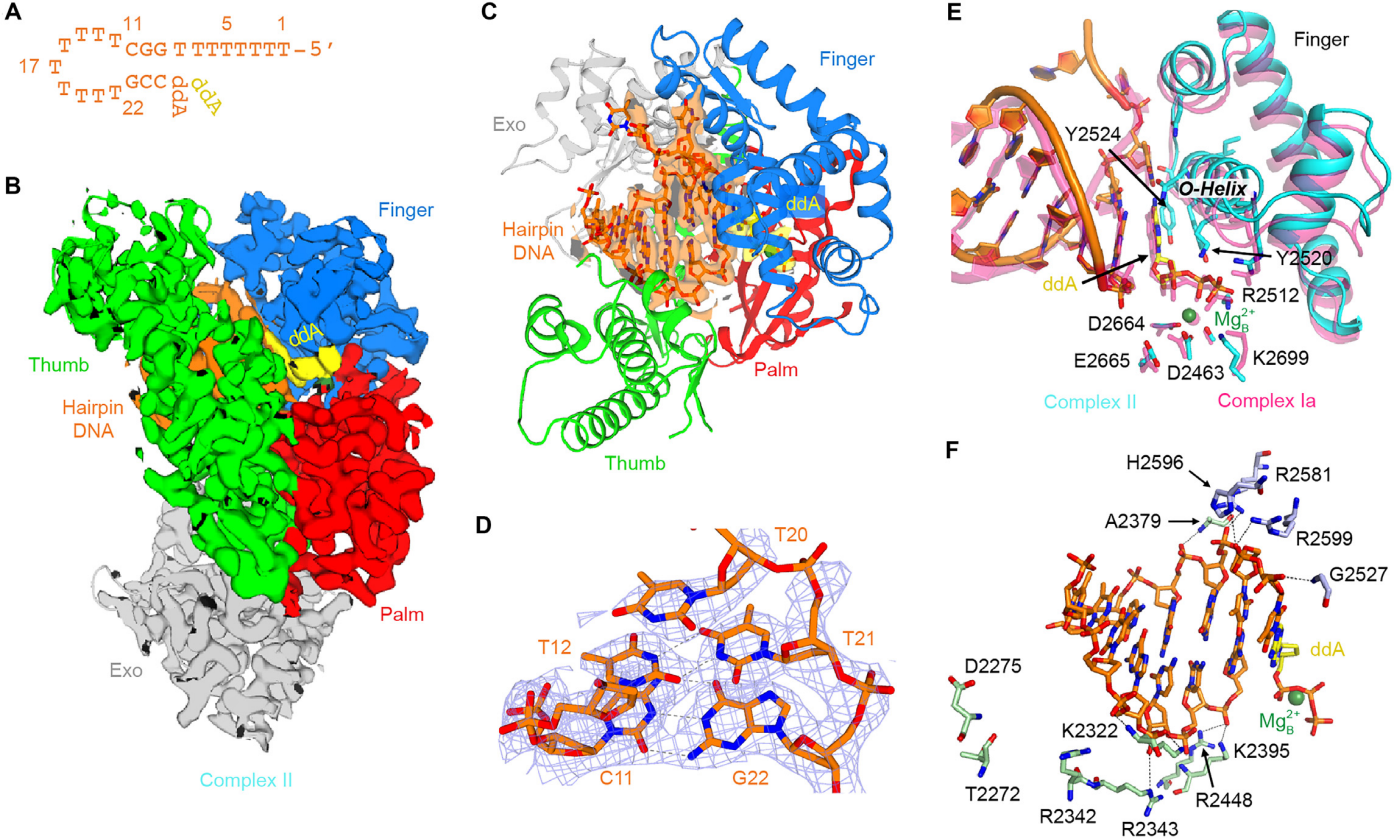
Cryo-EM structure of LcPol θ-pol complexed with a hairpin DNA. (**A**) The hairpin DNA substrate used for cryo-EM study. It is numbered from the 5′-end. (**B**) Overall cryo-EM structure of complex II at 3.0 Å. Individual domains are color-coded and the cryo-EM density map is overlaid upon the structural model. (**C**) A rotated view of complex II. Cryo-EM density map for the hairpin DNA is shown as orange surface. (**D**) Cryo-EM density map (mesh) of the hairpin DNA end. The model is shown as orange sticks. (**E**) Superimposition of the active sites of complexes Ia and II. Key residues and the O-helix are labeled. (**F**) Interactions between LcPol θ-pol and the hairpin DNA.

**Figure 5.**
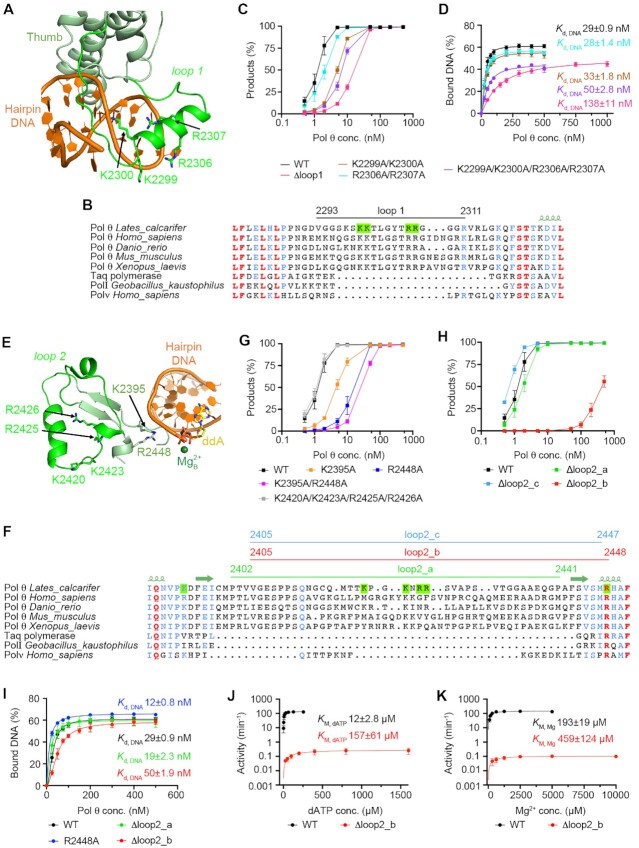
Computational modeling and mutagenesis studies of the insertion loops. (**A**) Predicted structure of the insertion loop 1 (florescent green) in the thumb domain (grey green). The conserved positively charged residues in LcPol θ loop 1 are shown as sticks. (**B**) Sequence alignment of A-family polymerases indicating the conservation of K2299, K2300, R2306 and R2307 (highlighted in green) in Pol θ and their absence in Taq, Pol I and Pol ν. (**C**) DNA synthesis activity of LcPol θ-pol with loop 1 deletion and mutations on the hairpin substrate HP [3,9]. (**D**) Binding affinity of WT and loop 1 mutants to HP [3,9]. (**E**) Predicted structure of the insertion loop 2 (florescent green) in the thumb domain (grey green). K2395 and R2448 in the flanking region of loop 2, and the positively charged residues in LcPol θ-pol loop 2 are shown as sticks. (**F**) Sequence alignment of A-family polymerases in the loop 2 region. Residues aforementioned in 5E are highlighted in green. Varied versions of loop 2 deletion are indicated with lines above the sequence alignment. (G, H) DNA synthesis activity of LcPol θ-pol with loop 2 mutations (**G**) and deletions (**H**) on HP [3,9]. (**I**) Binding affinity of WT and loop 2 mutants to HP [3,9]. (J, K) Steady-state kinetics of dATP incorporation (**J**) and Mg^2+^ utilization (**K**) by WT and Δloop2_b. All error bars represent the standard errors (*n* ≥ 3).

Steady state kinetics of WT and Δloop2_b was assayed as follows. For reactions shown in Figure 5J, 1 nM of WT LcPol θ-pol or 200 nM of Δloop2_b were mixed with 1 μM of HP [3,9] and incubated at 37°C for 5 min (WT) or 10 min (Δloop2_b) in 10 μl of reaction buffer containing 25 mM Tris [pH 7.6], 100 mM KCl, 3 mM DTT, 100 μg/ml BSA, 5% glycerol, 5 mM MgCl_2_ and varying concentrations of dATP (WT: 0, 2, 4, 8, 16, 32, 64, 128, 200 and 256 μM; Δloop2_b: 0, 25, 50, 100, 200, 400, 800, 1600 and 2000 μM). For reactions shown in Figure 5K, 1 nM of WT LcPol θ-pol or 400 nM of Δloop2_b were mixed with 1 μM of HP [3,9] and incubated at 37°C for 5 min (WT) or 10 min (Δloop2_b) in 10 μl of reaction buffer containing 25 mM Tris [pH 7.6], 100 mM KCl, 3 mM DTT, 100 μg/ml BSA, 5% glycerol, 100 μM dATP, and varying concentrations of MgCl_2_ (WT: 0, 100, 156, 200, 313, 625, 1250, 2500, 5000 and 10 000 μM; Δloop2_b: 0, 313, 625, 1250, 2500, 5000, 10 000, 12 500 and 15 000 μM). Reactions were quenched by the addition of 10 μl of the stop solution.

All reactions described above were analyzed by 20% denaturing polyacrylamide gel electrophoresis. Gels were visualized by Sapphire Biomolecular imager (Azure Biosystem), and bands were quantified with Azure Spot (Azure Biosystem). Steady state kinetics was fit to the model of ‘Michaelis–Menten equation’ using nonlinear regression in GraphPad Prism 9 to determine *K*_M_ and *k*_cat_. Multiple *t* tests were performed in Prism to obtain the *P* value at each enzyme concentration for each mutant in comparison to the WT. We considered *P* < 0.05 to be significant.

### Electrophoretic mobility shift assays (EMSA)

10 nM of HP [3,9] and increasing concentration of WT or mutant LcPolθ-pol (as indicated in Figure 5 and Supplementary Figure S10) were mixed and incubated on ice for 20 min in 20 μl of binding buffer containing 25 mM HEPES [pH 8.0], 100 mM KCl, 3 mM DTT, 100 μg/ml BSA, 10% glycerol, 2.5 mM EDTA and 0.01% NP40. Binding of LcPolθ-pol to HP [3,9] was analyzed by 6% native PAGE buffered with 1× Tris-glycine. Gels were visualized by Sapphire Biomolecular imager (Azure Biosystem), and bands were quantified with Azure Spot (Azure Biosystem). The binding results were fit to the model of ‘specific binding with Hill slope’ using nonlinear regression in GraphPad Prism 9 to determine the *K*_d_ values.

### Cryo-EM data collection

To prepare the ternary complex of LcPol θ-pol with dsDNA, 12 μM of the protein was first incubated with 16 μM of the 19/29 mer and 100 μM ddATP (ddA) at 25°C for 10 min in reaction buffer (25 mM Tris [pH 7.6], 100 mM KCl, 3 mM DTT and 5 mM MgCl_2_) to generate a ddA-terminating end. 1 mM dGTP was added to the reaction mixture and the incubation proceeded for another 10 min at 25°C. The complex of LcPol θ-pol with the hairpin substrate HP [3,9] was prepared in a similar way, but the protein-DNA mixture was supplied only with 100 μM ddA, which serves as the chain terminator at the end of the stem region and as the incoming nucleotide. 3.0 μl of freshly prepared complex sample was applied onto glow-discharged Quantifoil R1.2/1.3 Quantifoil holey carbon or UltrAuFoil holey gold R1.2/1.3 grids (Quantifoil). The grid was blotted for 4 s in 100% humidity at force –3 and was plunge-frozen in liquid ethane using a Vitrobot Mark IV (FEI).

### Cryo-EM data processing

CryoEM data were collected using Titan Krios (Thermo Fisher) transmission electron microscope, equipped with a K3 direct electron detector and post-column GIF (energy filter). K3 gain references were collected just before data collection. For LcPol θ-pol complexed with dsDNA, data collection was performed using SerialEM software (28) with image shift protocol (nine images were collected with one defocus measurement). Movies were recorded at defocus values from –0.8 to –1.6 μm at a magnification of 81k×, which corresponds to the pixel size of 1.06 Å at the specimen level (super-resolution pixel size is 0.53). During 3.6-s exposure, 60 frames (0.06 s per frame and the dose of 1.2012 e/frame/Å^2^) were collected with a total dose of 72 electrons Å^−2^. In total, 6409 images were collected. Prior to particle picking, micrographs were analyzed for good power spectrum, and the bad ones were discarded (with 6311 good images remained).

For LcPol θ-pol complexed with the hairpin substrate HP [3,9], data collection was performed in an automatic manner using EPU software (Thermo Fisher Scientific). Movies were recorded at defocus values from –1.0 to –2.0 μm at a magnification of 105k×, which corresponds to the pixel size of 0.826 Å at the specimen level (super-resolution pixel size is 0.413). During 3.0-second exposure, 60 frames (0.05 s per frame and the dose of 1.1103 e/frame/Å^2^) were collected with a total dose of 67 electrons Å^−2^. In total, 4357 images were collected. Prior particles picking micrographs were analyzed for good power spectrum, and the bad ones were discarded (with 4208 good images remained).

Data processing strategies were same for the two datasets. Motion correction was performed on raw super-resolution movie stacks and binned by 2 using MotionCor2 software (29). Contrast transfer function (CTF) estimation was performed using Gctf (30). Data processing was performed in cryoSPARC (31). Particles were selected using a template picker in cryoSPARC. Several rounds of 2D classification were performed to eliminate ice, carbon edges, and false-positive particles containing noise. Good 2D classes were selected, and ab initio reconstruction was performed to generate an initial reference map. Next, Heterogeneous Refinement and NU_refinement were performed iteratively to improve the resolution. Finally, local and global CTF refinement followed by NU_refinement or focused 3D refinement resulted in a resolution of 2.4 and 2.8 Å for the complexes Ia and Ib of LcPol θ-pol bound with dsDNA, respectively, and 3.0 Å for LcPol θ-pol bound with the hairpin substrate HP [3,9] using the 0.143 cutoff criterion (32). Local resolution was estimated in cryoSPARC.

### Model building and refinement

A homolog model of LcPolθ-pol was generated with the I-TASSER server (https://zhanglab.dcmb.med.umich.edu/I-TASSER/) and was used for initial rigid-body search. Each of the protein chains and the DNA were manually docked into the cryo-EM density maps in Chimera (33). The models were first manually adjusted in COOT (34) and then refined in Phenix (35) with real-space refinement and secondary structure and geometry restraints. Statistics of all cryo-EM data collection and structure refinement are shown in Table S1.

### Structure prediction

The loop modeling tools in Rosetta (36) were used for building the missing loops in complex II. The missing residues were first explicitly assigned in the blueprint file of remodel mode. This mode uses ref2015_cart score function to generate a total of 40 structures (37). For loop 1 modeling, structure that has the shortest distance between loop 1 and DNA was picked out. For loop 2 modeling, structure that has the center of mass of the loop nearest to the DNA backbone was picked out. Following that, a short all-atom simulation with the CHARMM36 force field was done to equilibrate the protein–DNA system. NAMD with 298 K fixed temperature and 1 ps timestep is applied to the 50 000 step molecular dynamics simulation (38). Structure of the last frame was picked out for analysis.

### Gel-filtration and crosslinking

Gel-filtration of LcPol θ-pol was performed using Superdex 200 at 4°C in buffer containing 25 mM Tris [pH 7.6], 250 mM KCl and 3 mM DTT. The composition of each peak was checked by SDS-PAGE. Crosslinking assay was carried out by treating varying concentrations of WT or mutant LcPol θ-pol with 0.1% glutaraldehyde in the absence of DNA or in the presence of equal amounts of HP [3,9] or MH6 (0.5, 1, 2 and 5 μM). The reactions proceeded for 5 min at 37°C in 50 μl of buffer containing 20 mM HEPES [pH 7.6], 100 mM KCl, and 3 mM DTT before quenching by the addition of 5 μl of 1 M Tris [pH 7.6]. Formations of LcPol θ-pol oligomers were visualized by SDS-PAGE.

## RESULTS

### Structure of Pol θ-pol with duplex DNA

LcPol θ (XP_018551255.1) is 2714 amino-acid long with an N-terminal hel domain, a C-terminal pol domain, and an unstructured central domain (Figure 1A). The hel and pol domains share 68% and 75% similarities to those of HsPol θ, respectively (Supplementary Figure S1). LcPol θ was chosen due to its high similarity to the human homolog and better yield and stability for biochemical and structural studies. Similar to HsPol θ-pol, LcPol θ-pol consists of four subdomains: the inactivated pseudo-exonuclease (residues 1975–2211), the thumb (residues 2212–2452), the finger (residues 2467–2608), and the palm (residues 2453–2466, 2609–2714), according to the common polymerase nomenclature. Five insertion loops are found in LcPol θ-pol, two in the pseudo-exonuclease domain (exo-loop 1, residues 2016-2048, and exo-loop 2, residues 2073–2094), two in the thumb domain (loop 1, residues 2288–2317, and loop 2, residues 2401–2442), and one in the finger domain (loop 3, residues 2639–2653). We constructed the polymerase domain of LcPol θ (LcPol θ-pol, residues 1950–2714) according to previous studies on HsPol θ and purified it to homogeneity (Figure 1B). To confirm the activity of LcPol θ-pol in DNA elongation and MMEJ, we performed DNA synthesis assays with LcPol θ-pol on a regular primer/template dsDNA (19/29 mer) and on a DNA substrate containing 6 bp of microhomology (MH6). Similar to HsPol θ-pol (3), LcPol θ-pol can robustly extend both regular dsDNA and MH6 to the end of the template, resulting in a +10 product (Figure 1C and 1D). A small portion of a +11 product was observed on both substrates at a higher concentration of LcPol θ-pol, which is likely due to the terminal transferase activity (39). Our biochemical assays confirmed that LcPol θ is a proper homolog of HsPol θ for mechanistic studies.

We sought to determine high-resolution structures of LcPol θ-pol with cryo-electron microscope (cryo-EM). To assemble the ternary complex, LcPol θ-pol was incubated with the incoming nucleotide dGTP, Mg^2+^, and a DNA duplex (20/29 mer) with a dideoxy-A (ddA)-terminating end, to prevent chain elongation. Despite its small size (99 kDa), the complex was with good contrast even on micrographs with low defocus, and the 2D-classification revealed high-resolution features (Supplementary Figure S2). Final 3D reconstruction and refinement yielded two structures at 2.4 Å (complex Ia) and 2.8 Å (complex Ib). For both complexes, the palm and finger domains have higher resolution compared to the pseudo-exonuclease and the thumb domains as well as the DNA ends. 11 and 18 bp of the duplex DNA are visible in complexes Ia and Ib, respectively. The high-resolution maps allow confident modeling of most protein side chains, DNA bases, Mg^2+^ ions and the incoming dGTP (Supplementary Figure S3). Nevertheless, the insertion loops remained mostly disordered (Supplementary Figure S3), similar to those in previous crystal structures of HsPol θ-pol (25).

Despite the difference in DNA length, protein portions of complexes Ia and Ib are similar with a RMSD of 0.6 Å based on alignments of Cα atoms (Figure 1E to 1G). In complex Ia, the DNA substrate around the minor groove contacts the thumb domain (Figure 1H). In contrast, the distal end of the duplex DNA in complex Ib shifts around 5 Å away from the thumb domain (Figure 1H). As a result, contacts between the template strand backbone and the thumb are lost, which is similar to that in HsPol θ-pol ternary complex (5). In addition, the H1- and H2-helices in both complexes Ia and Ib adopt a distinct conformation compared to those in HsPol θ-pol: the H1-helix in LcPol θ-pol moves towards the DNA backbone and the H2-helix rotates 40 degrees, resulting in K2322 contacting different parts of the DNA backbone. In a previously reported structure of HsPol θ-pol complexed with an RNA/DNA hydrid, the H1- and H2-helices appear as irregular loops (Supplementary Figure S4A) (26).

The finger domain in both complexes Ia and Ib exists in a closed state with the incoming dGTP and one Mg^2+^ ion bound to the active site. Interestingly, conformation of the finger domain in complexes Ia and Ib is distinct from that in the previous HsPol θ-pol structures (Figure 1I). The finger domain rotates 30 degrees towards the active center compared to the open finger state in the HsPol θ-pol-RNA/DNA structure (Supplementary Figure S5A), and rotates 7 degrees downward to the triphosphate group of the incoming nucleotide compared to the closed finger state in the HsPol θ-pol-dsDNA structure (Figure 1I). The end of the O-helix, which has been shown to play a critical role in closing the active sites of A-family polymerases, shifts 5 Å and 8 Å compared to that in the closed and the open complexes of HsPol θ-pol, respectively (Figure 1I and Supplementary Figure S5A). In the recently reported HsPol θ-pol inhibitor structure, the finger domain also takes a partially closed state compared to that in complex Ia (Supplementary Figure S5B). Consequently, R2512 in complex I forms proper contacts with the gamma-phosphate group, and the dGTP shifts 1.5 Å to the Mg^2+^ binding site. R2512 is highly conserved among A-family polymerases; mutating it to an alanine results in severe loss of polymerase activity (40). Our closed finger conformation of LcPol θ-pol is similar to the well-defined closed state of Taq polymerase and Pol I (Supplementary Figure S5C) (41,42).

### Active site and DNA interaction

The 2.4 Å structure of the LcPol θ-pol complex Ia allows confident modeling of the active site (Figure 2A). The B-site Mg^2+^ is coordinated by three oxygen atoms on the incoming dGTP, D2463, D2664, and the backbone of Y2464 (Figure 2A and Supplementary Figure S3C). Due to the lack of the 3′-OH at the ddA-terminating end, the A-site, which should be formed by D2664, D2463 and E2665, shows no density (Figure 2A). The finger domain movement brings R2512, K2516 and Y2520 to contact the triphosphate group of dGTP. K2699 and the backbone of Q2466 interact with the gamma-phosphate. The incoming dGTP forms a Watson–Crick base pair with the nascent template dC and base-stacks on the primer end. Backbones of residues 2520–2524 within the O-helix sit on top of the nascent base pair. The active site and the nascent base pair are almost superimposable to the ternary complex of Taq (Supplementary Figure S5D). The nascent base pair is surrounded by a series of hydrophilic residues, with Q2534, Q2517, and K2516 on one side, and R2382, Q2607 and N2603 on the other side (Figure 2B). These residues may stabilize abnormal base pairs during TLS, as shown in the case of low-fidelity Y-family Pol η (43). Among them, Q2517 is the most divergent residue in A-family polymerases, with T and K in Taq and Pol ν, respectively, and Q in Hs and LcPol θ. It has been shown that Q2517 in HsPol θ helps to stabilize the incoming dATP against an AP lesion (25). Moreover, mutating the corresponding K in HsPol ν enhances its fidelity (44).

LcPol θ-pol forms tight interactions with the first 2–3 bp of the DNA substrate at the elongation site (Figure 2C). Specifically, residues K2395 and R2448 from the flanking region of the disordered loop 2 contact the primer end. For the template strand, side chains of R2599, H2596, R2581 and Q2600 and the mainchain of A2379 interact with the DNA backbone of the first two nucleotides while G2527 from the Oα-Helix stabilizes the kink of the template. Compared to Taq, the thumb domain of LcPol θ-pol adopts a similar conformation (Supplementary Figure S4B); however, LcPol θ-pol exhibits limited interactions with the distal-end DNA (Figure 2D and Supplementary Figure S4B). K2322 adopts a distinct conformation from that in HsPol θ-pol, which uses its backbone and side chain to interact with T17 and A18 on the primer. In complex Ia, the thumb domain forms contacts with the duplex DNA substrate mainly at its minor groove. R2342, K2322 and R2343 interact with the primer backbone, and T2272 and D2275 are in proximity to the template backbone. Although the thumb-primer interactions are similar in complex Ib, the template strand is too far away to directly contact the thumb domain (Figure 1H).

### Design and characterization of hairpin DNA substrates

The key feature of MMEJ substrates lies in the short (2–6 bp) microhomologous duplex. However, obtaining stably annealed 2–6 bp duplexes for Pol θ structural study is technically challenging. Given that Pol θ can extend DNA ends with transiently formed hairpins within the same strand during MMEJ (5,13), we designed hairpin DNA structures to mimic MMEJ substrates. The hairpin substrates contain a short duplex stem, a poly-dT hairpin loop, and a 5′ overhang of eight dTs (Figure 3A). We first tested whether the number of base pairs in the stem region affected LcPol θ-pol activity. With 2-bp stem, LcPol θ-pol extended small portions of the hairpin substrates: 23% to the end of the templating region (+8) and 8% to +9 (Figure 3B), as the product of its terminal transferase activity (39 ). With 3-bp stem, a dramatic increase in LcPol θ-pol performance was observed, evidenced by the fact that nearly all substrates were consumed at 10 nM enzyme. With 4–6 bp stem, full substrate conversion was observed at 5 nM enzyme. For all substrates, the +9 products were observed mostly at 50 nM of LcPol θ-pol, the highest concentration examined. To confirm that LcPol θ-pol incorporated the correct nucleotides during hairpin extension, we checked the fidelity of LcPol θ-pol with individual nucleotides. As expected, the hairpin substrate was extended to full products only when dATP was provided (Figure 3C). A small amount of +1 product was observed with dTTP and dGTP, consistent with the previous report that HsPol θ-pol misincorporates dTTP and dGTP opposite a templating T (45). These results suggest that LcPol θ-pol can efficiently utilize the hairpin substrates with 3-bp or longer stems for template-dependent DNA synthesis, a pattern similar to those of hetero-duplex MMEJ substrates (3).

We next examined whether the length of the hairpin loop affected LcPol θ-pol activity, as in vivo assays indicated that Pol θ was able to find microhomology regions within a strand over a long distance (4). With the stem region fixed to 3 bp, we designed substrates with the loop length ranging from 3, 6, 9, 12 to 15 nt. The activity of LcPol θ-pol increased with longer loop length from 3 nt to 9 nt, as nearly full substrate consumption (93%) was achieved for HP [3,9] at 1 nM of LcPol θ-pol (Figure 3D). At higher concentrations of LcPol θ-pol, more substrates and/or intermediates were converted to +8 and +9 products. When the loop length further increased to 12 and 15 nt, less substrate usage was observed at 1 nM of LcPol θ-pol (88% and 57%, respectively) compared to the 9 nt-loop. The above data suggests that these hairpin DNA structures are proper mimics of MMEJ substrates for LcPol θ-pol. We used the hairpin substrate with 3-bp of microhomology and a 9-nt loop (HP [3,9]) for subsequent structural and mechanistic investigations.

### Structure of Pol θ-pol with a hairpin substrate

To construct the ternary complex of LcPol θ-pol with the hairpin substrate, ddA was supplied both as the chain terminator at the end of the stem region and as the incoming nucleotide (Figure 4A). With cryo-EM, we obtained a 3.0 Å resolution structure of LcPol θ-pol complexed with the hairpin DNA HP [3,9], ddA, and Mg^2+^ (complex II) (Supplementary Figure S6). The overall structure is similar to those bearing long duplex DNA, with a RMSD of 1.1 Å to complex Ia (Figure 4B). The stem of the hairpin DNA is clearly visible within the DNA binding site (Figure 4C). Interestingly, although only 4 base pairs (3 bp + ddA-T) should be present as designed, we observed additional density covering the DNA end. Two dTs form a mismatched base pair and an additional dT is stacked on top of the dT-dT mismatch (Figure 4D). Engagement of DNA mispairing and the additional base may help in stabilizing the short microhomologous region during extension.

Conformation of the hairpin DNA in complex II is superimposable to the long duplex DNA in complex Ia (Figure 4E). At the proximal end, H2596, R2581, R2599 interact with the nascent template, G2527 stabilizes the kink of the template, and K2395 and R2448 form salt links with the primer end (Figure 4F). The H2-helix adopts the same orientation as in complex Ia, with K2322 interacting with the DNA substrate using both its side chain and backbone. R2342, D2275 and T2272, on the other hand, show no contact to the hairpin due to the shorter DNA length. Consequently, the H1 helix in complex II rotates 10° relative to that in complex Ia (Supplementary Figure S7A). The presence of ddA and Mg^2+^ is confirmed with their corresponding cryo-EM densities (Supplementary Figure S7B). However, the finger domain is in a less closed state compared to that in complex Ia and rotates 3 degrees up away from the active site (Supplementary Figure S7C). The O-helix with Y2524 and Y2520 still stacks near the nascent template-incoming nucleotide base pair and R2512 interacts with the gamma-phosphate (Figure 4E). Yet, the alpha-phosphate is lifted around 1 Å and the B-site Mg^2+^ coordination becomes less favorable (Supplementary Figure S7D).

### Modeling of disordered loops in the thumb domain

Previous biochemical studies suggested that insertion loop 1 is important for the processivity of HsPol θ-pol (16). Sequence alignment showed that loop 1 in Pol θ is much longer than the corresponding loops in Taq and Pol I (Figure 5B), which have been shown to interact with DNA substrates (41,43) (Supplementary Figure S4B). However, loop 1 remains disordered in all complexes Ia, Ib and II, similar to previous crystal structures of HsPol θ-pol. To obtain mechanistic insights into loop 1 function, we performed structural prediction with a Rosetta loop modeling tool (Supplementary Figure S8A), followed by the all-atom molecular dynamics (MD) simulation. The final model suggests that loop 1 is located on top of the hairpin DNA (Figure 5A), with four positively charged residues, K2299, K2300, R2306, and R2307, interacting with the DNA backbone. These residues are conserved in Pol θ but are absent in Pol ν, Taq and Pol I (Figure 5B). To confirm the roles of these positively charged residues, we constructed a LcPol θ-pol loop 1-deleted mutant (Δloop1, Δ2293–2311), and three variants of K2299A/K2300A, R2306A/R2307A and K2299A/K2300A/R2306A/R2307A. DNA synthesis assays on the hairpin substrate HP [3,9] with these mutants showed that loop 1 deletion significantly reduced the activity of LcPol θ-pol (Figure 5C and Supplementary Figure S9A). Over 100 nM of Δloop1 were needed to achieve full substrate consumption, whereas 10 nM of WT LcPol θ-pol were enough to convert all substrates. Similarly, K2299A/K2300A and R2306A/R2307A showed decreased activity relative to the WT enzyme, indicating the importance of these positively charged residues. Of interest, substituting all four positively charged residues (K2299A/K2300A/R2306A/R2307A) further reduced the DNA synthesis activity relative to the double mutants, suggesting an additive effect for these residues. DNA binding assays confirmed that deletion of loop 1 drastically reduced the capability of LcPol θ-pol binding to the hairpin substrate (*K*_d_ = 138 nM) relative to the WT (*K*_d_ = 29 nM) (Figure 5D and Supplementary Figure S10). In contrast, K2299A/K2300A and R2306A/R2307A bind to the hairpin substrate with similar affinity as the WT enzyme. Nevertheless, K2299A/K2300A/R2306A/R2307A showed a lower binding affinity with a *K*_d_ value of 50 nM. It is possible that these four residues work in complementary during short DNA binding; mutating all of them at the same time decreases Pol binding affinity. Taken together, we conclude that loop 1 stabilizes the binding of Pol θ to short duplex DNA and possibly orient them for DNA synthesis during MMEJ. We want to point out that a single model may not picture the whole scenario of how loop 1 functions during MMEJ, especially because it is highly flexible and may adopt several conformations during different stages of DNA synthesis. As in Figure 5C, mutation of all four positively charged residues is less detrimental than Δloop1, suggesting that additional structural elements, possibly protein backbones or other sidechains, may contribute to DNA binding and orientation as well when loop 1 takes other conformations. Nevertheless, the model presented here could still help to target important residues and depict the transient interaction between loop 1 and the DNA substrate.

Loop 2 is known to be critical for activities of HsPol θ in MMEJ and TLS (5,16). The hinge of loop 2 forms a beta-hairpin structure, with K2395 and R2448 interacting with the backbone of the primer end (Figure 2C). However, the majority of loop 2 remains disordered in our structures, similar to previous crystal structures of HsPol θ-pol (25). To examine the function of loop 2, we constructed a loop 2 model with similar Rosetta modeling and MD simulations. Neither the Rosetta models nor structures during MD simulation showed that loop 2 approached the active site (Figure 5E and Supplementary Figure S8B). We noticed that there were positively charged clusters in loop 2 in both Hs and LcPol θ (Figure 5F), though not as conserved as those in loop 1. Mutating all of them to alanine (K2420A/K2423A/R2425A/R2426A) exhibited minimal effect on LcPol θ-pol DNA synthesis activity for HP [3,9] (Figure 5G and Supplementary Figure S9B). In contrast, both single mutations of K2395A and R2448A at the hinge of loop 2 showed substantially decreased DNA synthesis. The equivalent residue of K2395 in HsPol θ-pol, R2254, has been indicated as the starting point of loop 2 and mutating it to alanine or valine slightly reduces HsPol θ-pol DNA synthesis activity on a regular primer/template dsDNA (25). Double mutation of K2395 and R2448 to alanine (K2395A/R2448A) further decreased LcPol θ-pol activity (Figure 5G). To better understand the role of loop 2, we designed a series of loop 2 deletions (Figure 5F). Δloop2_a (ΔP2402-P2441) was able to extend HP [3,9] as robustly as the WT enzyme (Figure 5H and Supplementary Figure S9B), which differs from previous findings for HsPol θ-pol (5,16). To investigate this inconsistency, we designed Δloop2_b (ΔV2405–R2448) that is equivalent to the HsPol θ_Δloop2 in literature (5,16) (Figure 5F). Similar to HsPol θ_Δloop2, Δloop2_b exhibited severe inhibition in extending HP [3,9] (Figure 5H). A third loop 2-deleted mutant (Δloop2_c, ΔV2405–M2447), which restores R2448 to Δloop2_b, showed DNA synthesis activity comparable to WT LcPol θ-pol, confirming the critical role of R2448 in DNA extension (Figure 5H). Interestingly, Δloop2_a, Δloop2_b, and R2448A are all able to bind the hairpin substrate HP [3,9] as efficiently as WT LcPol θ-pol with comparable *K*_d_ values (Figure 5I and Supplementary Figure S10). In contrast, steady-state kinetics of dATP incorporation and Mg^2+^ utilization showed a 13-time higher *K*_M, dATP_ (157 μM) and a 2.4-time higher *K*_M, Mg_ for Δloop2_b (459 μM) relative to WT LcPol θ-pol (*K*_M, dATP_ 12 μM; *K*_M, Mg_ 193 μM), respectively (Figure 5J, K and Supplementary Figure S11). The weaker binding capability of Δloop2_b for the incoming dATP and Mg^2+^ explains the decreased activity of Δloop2_b in DNA synthesis. To conclude, our computational modeling and mutagenesis studies suggest that loop 2 contributes to primer alignment and active site assembling with its positively charged residues in the hinge, whereas the major portion of loop 2 is not essential for Pol θ activity.

Previous reports have proposed a dimer model for HsPol θ-pol based on crystal lattice contact (25), and loop 2 has been suggested to engage in the dimer formation (5). However, no dimer or oligomer formations of LcPol θ-pol were found in the 2D-classification during cryo-EM data processing (Supplementary Figure S2A), indicating weak or transient interactions. Gel filtration of LcPol θ-pol showed that the protein is mainly monomeric with a small peak possibly corresponding to the dimer (Supplementary Figure S12A, B). To further examine the oligomerization state of LcPol θ-pol and the function of loop 2, we performed in-solution crosslinking in the absence and presence of different DNA substrates. Without DNA, LcPol θ-pol could not be efficiently crosslinked even at 5 μM of the protein. In contrast, the presence of the hairpin (HP [3,9]) or MMEJ substrates (MH6) stimulated LcPol θ-pol crosslinking, resulting in dimeric, trimeric and higher-order oligomers on SDS-PAGE (Supplementary Figure S12C). Δloop2_c exhibited DNA-stimulated oligomerization (Supplementary Figure S12D) similar to WT LcPol θ-pol, indicating that loop 2 is not critical for Pol θ oligomerization.

## DISCUSSION

DNA polymerases usually interact with 10-bp or longer duplex DNA for DNA synthesis. The thumb domain plays critical roles in DNA binding. In A-family polymerases, the thumb domain contacts the DNA phosphate backbone near the first minor groove of both primer and template strands (Supplementary Figure S4). Moreover, replicative A-family polymerases are evolved to have long insertion loops and recruit additional subunits to interact with DNA backbones at the second minor groove (Supplementary Figure S4C, D). Nevertheless, replicative polymerases, such as T7 gp5, can still utilize short duplex during primer hand-off from primase to polymerase, where the Zinc-binding domain of the primase binds to the DNA binding channel of the polymerase and stacks at the DNA end to stabilize the short duplex for polymerase extension (46). Pol θ is a unique A-family polymerase in that it can utilize short duplex DNA for DNA extension without external factors, an activity essential for MMEJ. Our structures suggest that Pol θ binds to short and long duplex DNA in a similar manner. Consistent with that, interactions between the thumb domain and the template strand in Pol θ are weak or absent even in the presence of long duplex DNA (Figure 2D). Instead, Pol θ tightly holds DNA at its proximal end with numerous positively charged residues including those from the hinge of insertion loop 2 (Figure 5E). According to the results from mutagenesis studies and MD simulation, insertion loop 1 stacks at the DNA end with its positively charged side chains and further stabilizes the binding of Pol θ to the short duplex DNA (Figure 5A, D), possibly serving a similar function to the primase Zinc-binding domain during primer hand-off. In addition, we observed a dT-dT base pair with an extra dT stacking on top of the mismatch at the end of the short duplex. The mispairing and base stacking may further stabilize the short duplex binding and explain how Pol θ tolerates mismatches and internal microhomolous regions during MMEJ. Our studies suggest that the unique MMEJ activity of Pol θ is promoted by tight interactions between Pol θ and proximal DNA, the insertion loops, and possibly mispairing of DNA adjacent to the short microhomologous region.

Despite being a homolog to high-fidelity replicative A-family polymerases, the fidelity of Pol θ is comparable to that of low-fidelity Y-family polymerases (6). Additionally, Pol θ can perform efficient TLS across various DNA lesions (16–20), which is distinct from other A-family members. Previous extensive studies suggest that all DNA polymerases share similar Mg^2+^ organized active sites and catalytic mechanism (46). For TLS polymerases, pre-formed and enlarged active sites help to accommodate bulky lesions, while hydrophilic protein side chains help to stabilize unusual base pairs (Supplementary Figure S5F, G) (46). Our atomic resolution structures of Pol θ define details of its active site. The finger domain of Pol θ exists in a closed conformation superimposable to that of the high-fidelity Taq polymerase. The nascent base pair is found to be surrounded by hydrophilic residues, which may contribute to the stabilization of mismatched base pairs. The closed finger domain in Pol θ brings the O-helix right on top of the nascent base pair, leaving no room for bulky lesions or buckled base-pairing. On the other hand, the finger domain is highly flexible and can rotate in a variety of directions, as shown in our structures and previously determined HsPol θ-pol structures. The flexible finger domain and the hydrophilic side chains may allow Pol θ to tolerate mismatched and bulky lesions, such as CPD (17).

Pol θ bridges two ssDNA ends to perform DNA synthesis during MMEJ. Pol θ-hel has been shown to exist as a tetramer in crystal, but in that structure only the DNA ends bound to two protomers are close enough for synapsis formation (47). Previously, a dimer model of Pol θ-pol has been proposed to facilitate MMEJ based on crystal packing. However, no dimer or oligomer species of LcPol θ-pol was observed in cryo-EM (Supplementary Figure S2). The structure with short duplex DNA suggests that one LcPol θ-pol monomer is sufficient to bind to the short dsDNA and to form a proper ternary complex for DNA synthesis. In-solution crosslinking suggests that LcPol θ-pol can assemble into oligomers in the presence of DNA (Supplementary Figure S12). It is possible that Pol θ-pol form oligomers due to Pol θ-hel oligomerization and non-specific interactions between Pol θ-pol and DNA to promote efficient synapsis formation, while one Pol θ-pol is sufficient during MMEJ DNA synthesis. Once DNA is extended long enough, a second Pol θ-pol can catch the other free 3′-DNA end to perform DNA extension in the opposite direction.

In conclusion, our high-resolution structures of Pol θ with different DNA substrates illustrate the unique structural features of Pol θ for its low fidelity MMEJ synthesis. These findings may help in defining potential druggable sites and facilitating drug screening and optimization targeting Pol θ.

## ACCESSION NUMBERS

The cryo-EM density maps for complexes Ia, Ib and II have been deposited in the Electron Microscopy Data Bank under accession numbers EMD-28075, EMD-28078 and EMD-28084, respectively. The atomic coordinates of complexes Ia, Ib and II have been deposited in the Protein Data Bank under accession numbers 8EF9, 8EFC and 8EFK.

## Supplementary Material

gkac1201_Supplemental_File
