# Supplementary material for: Structural basis of DNA polymerase θ mediated DNA end joining
Source: Nucleic Acids Res. 2022 Dec 30;51(1):463–74. doi: 10.1093/nar/gkac1201 (PMC9841435; doi:10.1093/nar/gkac1201)
Supplement: gkac1201_Supplemental_File [file gkac1201_supplemental_file.pdf]

Supporting Information for

Structural basis of DNA polymerase θ mediated DNA end joining

Chuxuan Li, Hanwen Zhu, Shikai Jin, Leora M. Maksoud, Nikhil Jain, Ji Sun, and Yang Gao

Yang Gao
E-mail: yg60@rice.edu

This PDF file includes:

Figs. S1 to S12
Tables S1 to S2

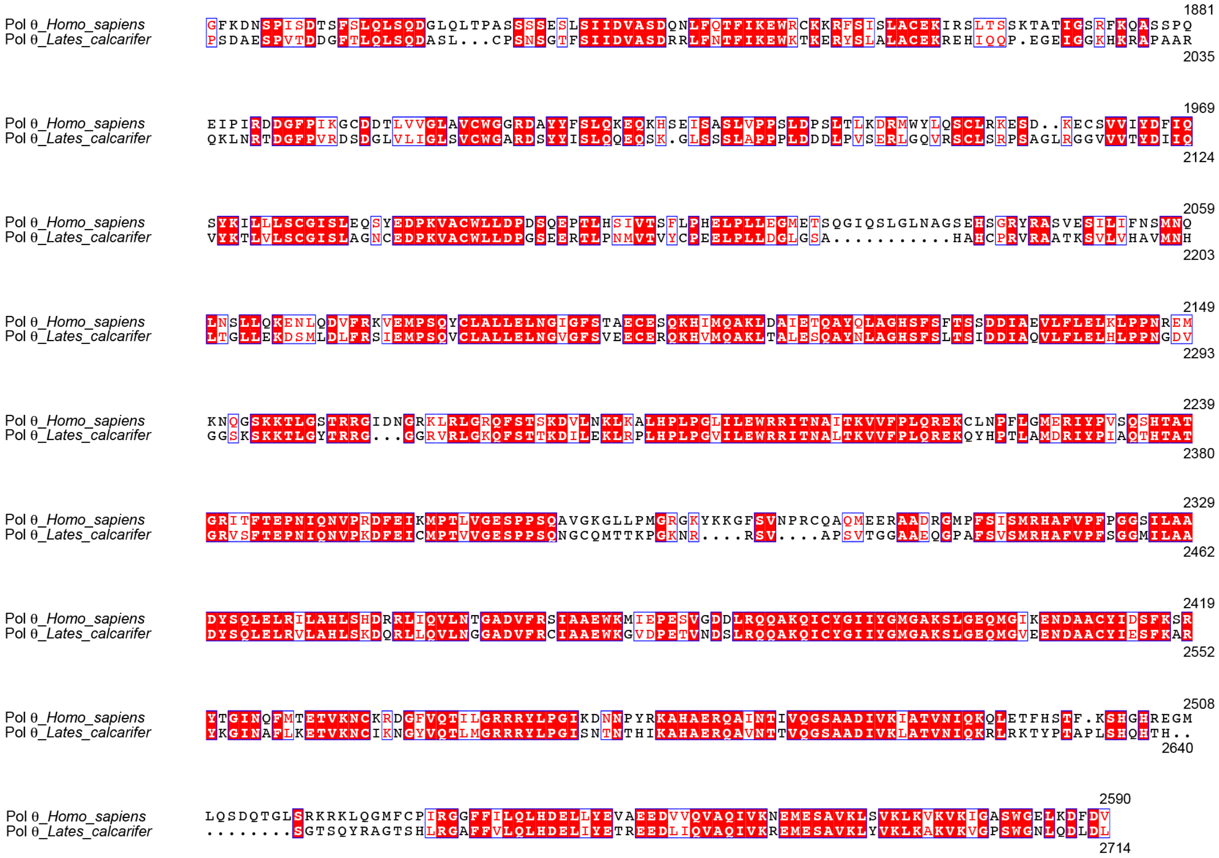

Fig. S1. Sequence alignment of LcPol θ-pol and HsPol θ-pol. The sequence alignment is performed with ESPript 3.0.

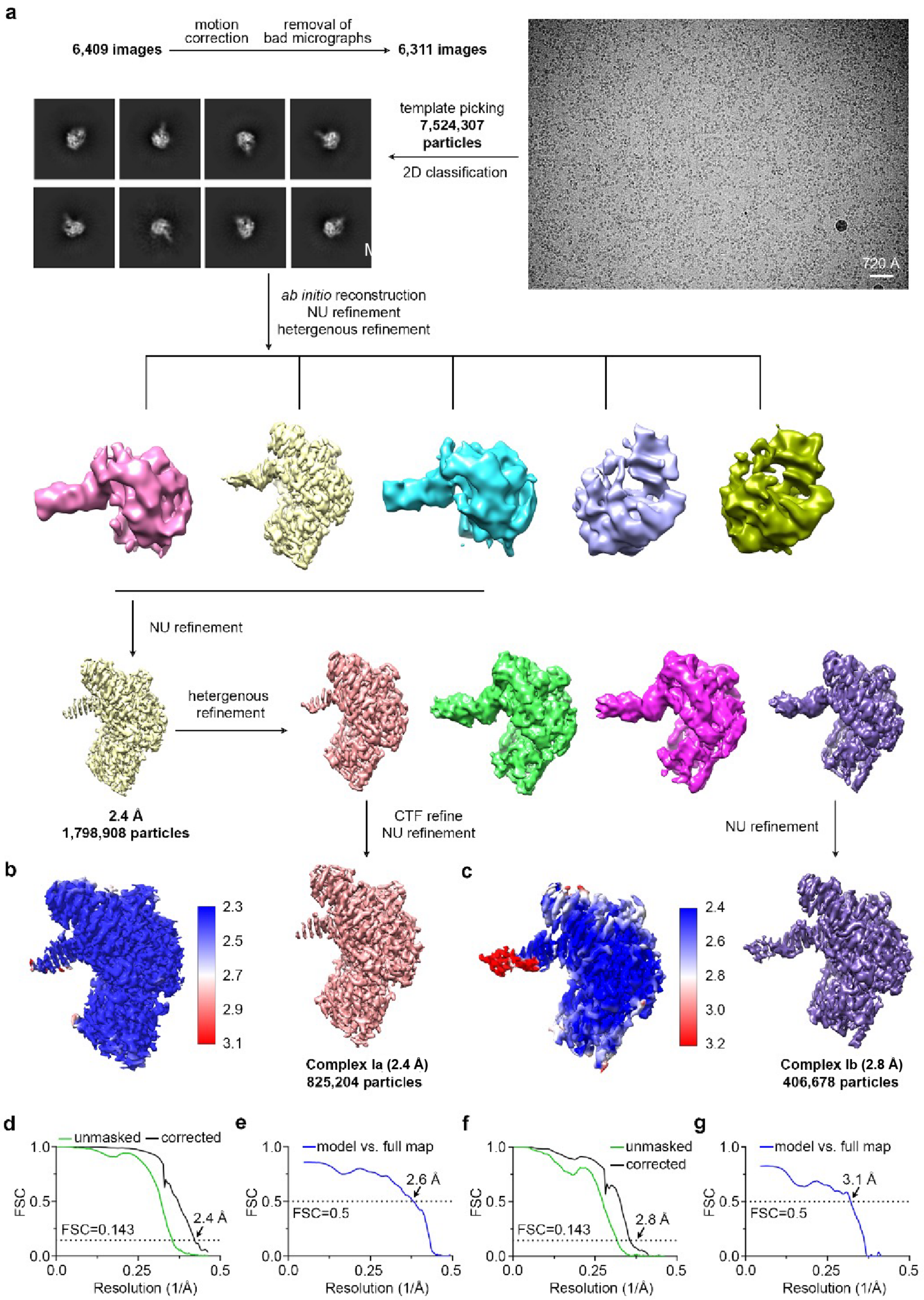

Fig. S2. Cryo-EM data processing scheme of LcPol θ-pol complexed with long dsDNA. **(a)** A simplified flow chart of the cryo-EM data processing. **(b, c)** Local resolution of complexes Ia and Ib. **(d, f)** Fourier Shell Correlation (FSC) curves for the overall resolution of complexes Ia and Ib. The golden standard (FSC=0.143) is used for resolution estimation. **(e, g)** Model-to-map fit between the full maps and PDB coordinates of complexes Ia and Ib, respectively.

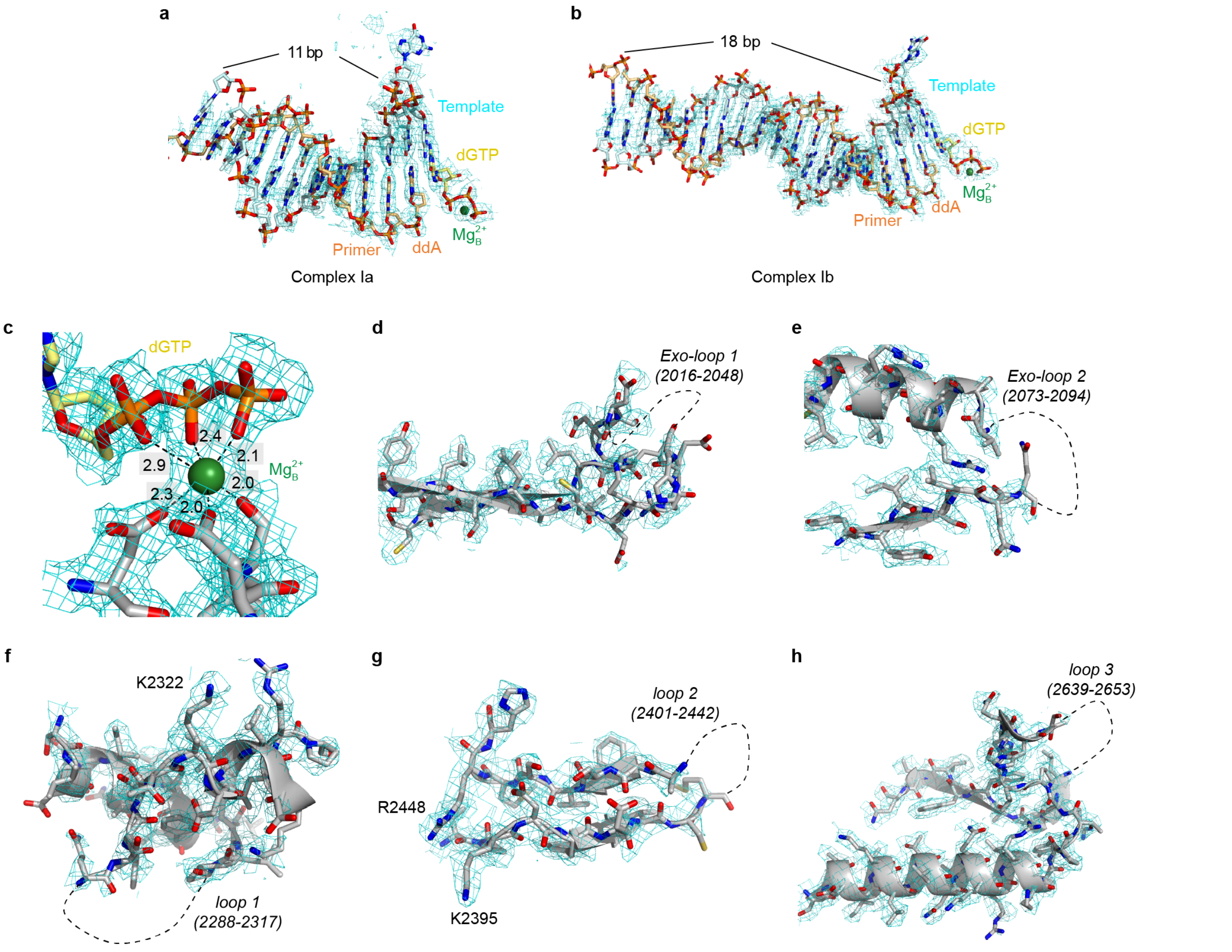

Fig. S3. Local cryo-EM density maps of complexes Ia and Ib. **(a, b)** Local cryo-EM maps for DNA in complexes Ia **(a)** and Ib **(b)**. **(c)** Local cryo-EM maps for the Mg^{2+} binding site in complex Ia. **(d-h)** Local cryo-EM maps for regions near the five insertion loops.

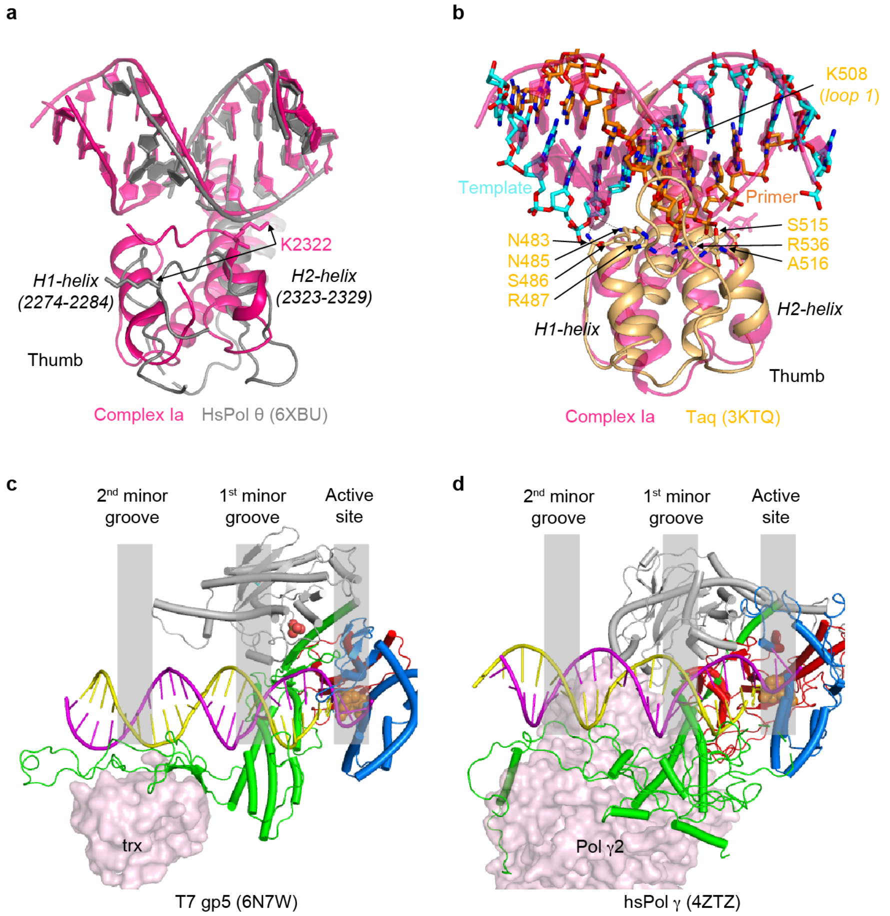

Fig. S4. DNA interactions in A-family DNA polymerases. **(a)** Structure superimposition of the thumb domains in complex Ia and HsPol θ-pol bound with an RNA/DNA hybrid (6XBU). **(b)** Structure superimposition of the thumb domains in complex Ia and Taq polymerase (3KTQ). Key residues in the Taq thumb domain for DNA interactions are indicated. **(c, d)** Structures of the replicative A-family polymerases from bacteriophage T7 **(c)** and human mitochondria **(d)**. The extended thumb loop (green) and the associated processivity factors (pale pink) are shown.

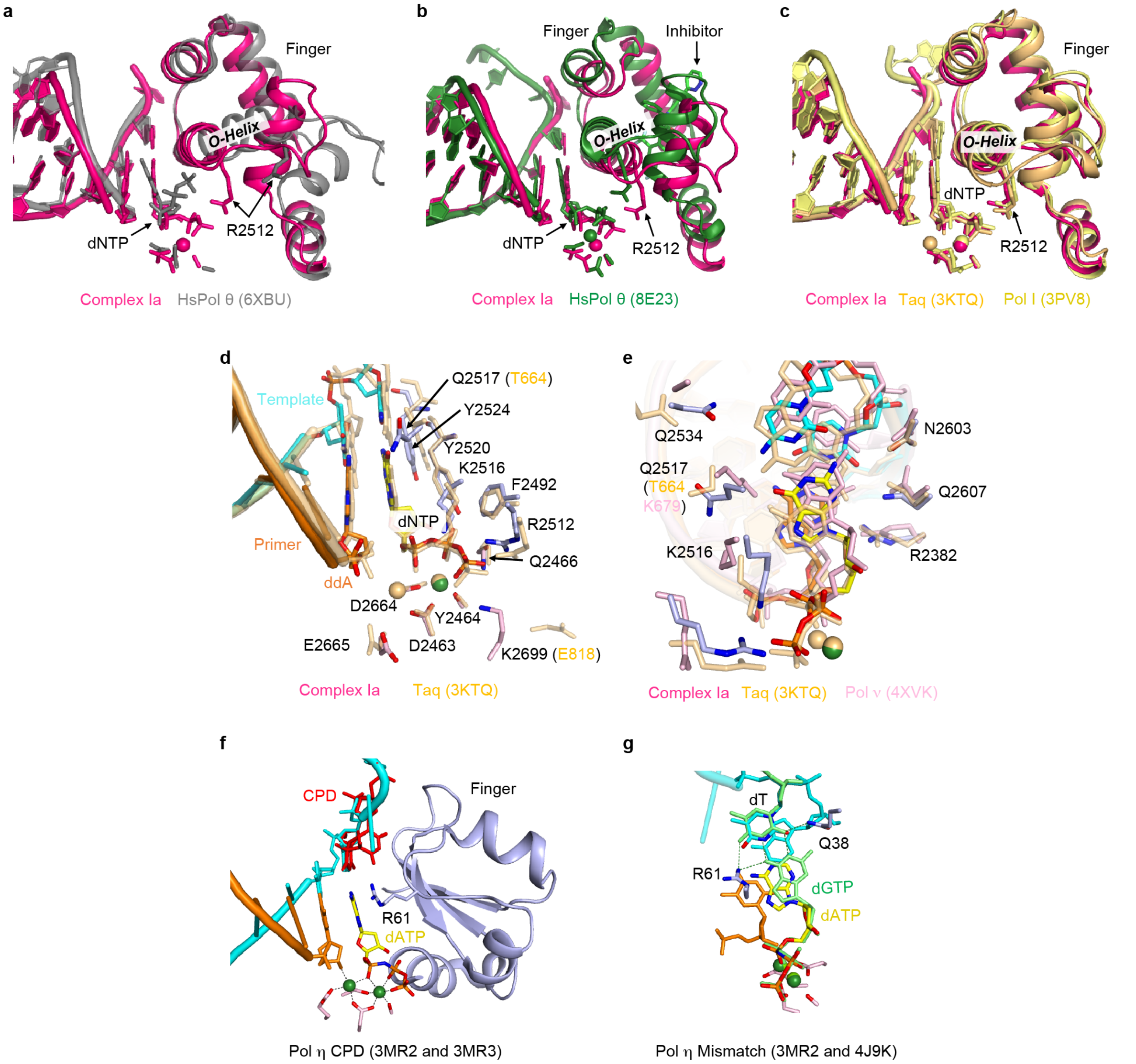

Fig. S5. Active site features of high- and low-fidelity DNA polymerases. **(a, b)** Structure superimposition of the finger domains in complex Ia and HsPol θ-pol bound with an RNA/DNA hybrid **(a)** or with undamaged duplex DNA in the presence of an inhibitor **(b)**. **(c)** Structure superimposition of the finger domains in complex Ia, Taq polymerase and Pol I suggests that complex Ia has a properly closed finger domain. **(d)** Structure superimposition of the active sites of complex Ia and Taq polymerase. **(e)** Comparison of the environment around the nascent base pair in Taq, Pol ν, and LcPol θ-pol (complex Ia). **(f, g)** Structure comparisons of Y-family Pol η incorporating against an undamaged base (cyan, 3MR2) and a CPD lesion (red, 3MR3) **(f)**, and inserting a mismatched nucleotide (light green, 4J9K) **(g)**. Key residues in the active sites are shown in sticks and labeled.

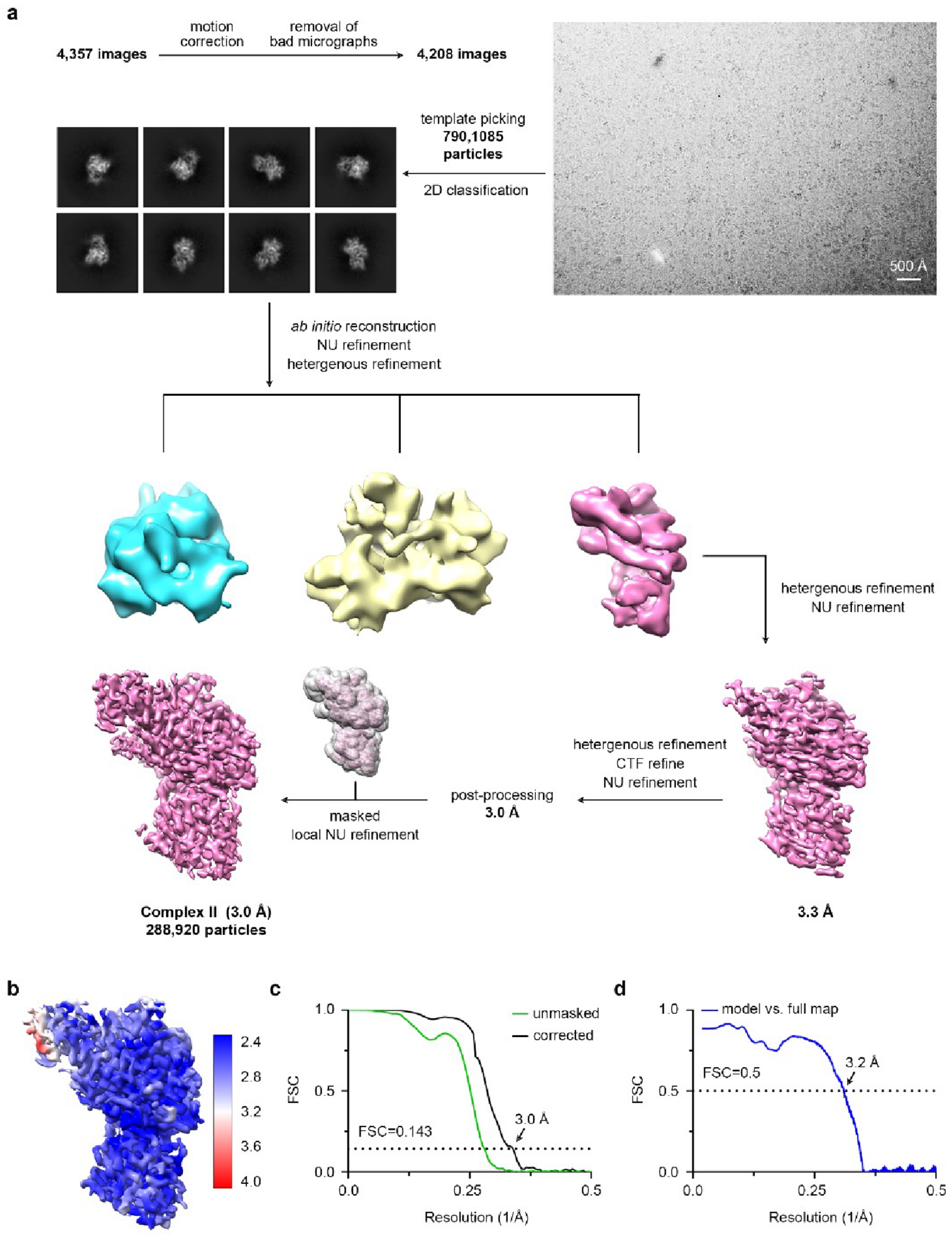

Fig. S6. Cryo-EM data processing scheme of LcPol θ-pol complexed with the hairpin DNA. **(a)** A simplified flow chart of the cryo-EM data processing. **(b)** Local resolution of complex II. **(c)** Fourier Shell Correlation (FSC) curves for the overall resolution of complex II. The golden standard (FSC=0.143) is used for resolution estimation. **(d)** Model-to-map fit between the full map and PDB coordinate of complex II.

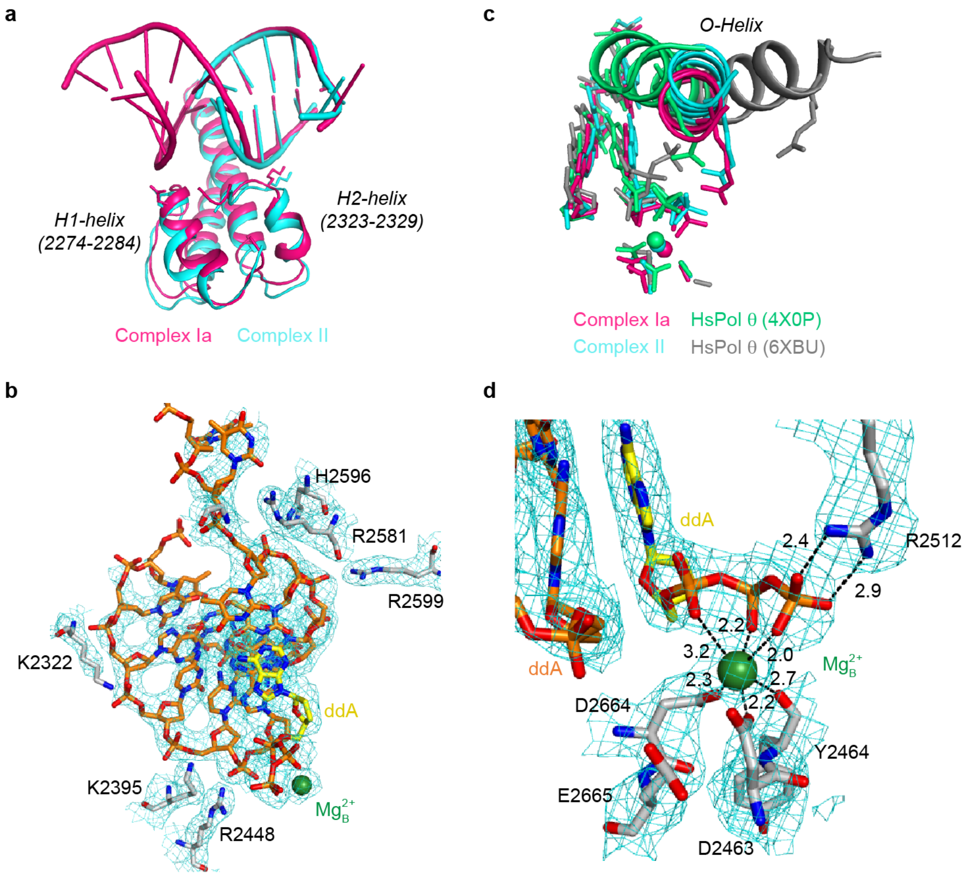

Fig. S7. Structural features of LcPol θ-pol complexed with the hairpin DNA. **(a)** Structure superimposition of the thumb domains in complexes Ia and II. **(b)** Local cryo-EM map for the DNA binding site in complex II. **(c)** Finger domain movement in complexes Ia and II comparing to that in HsPol θ-pol bound with a dsDNA (4X0P) or an RNA/DNA hybrid (6XBU). **(d)** Local cryo-EM map for the active site.

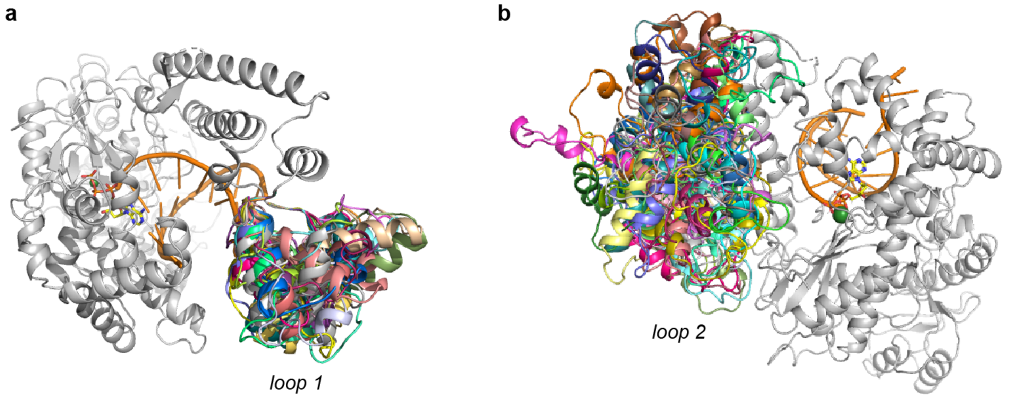

Fig. S8. Rosetta models of insertion loop 1 **(a)** and loop 2 **(b)** in the thumb domain. Forty models of loop 1 and loop 2 are overlaid.

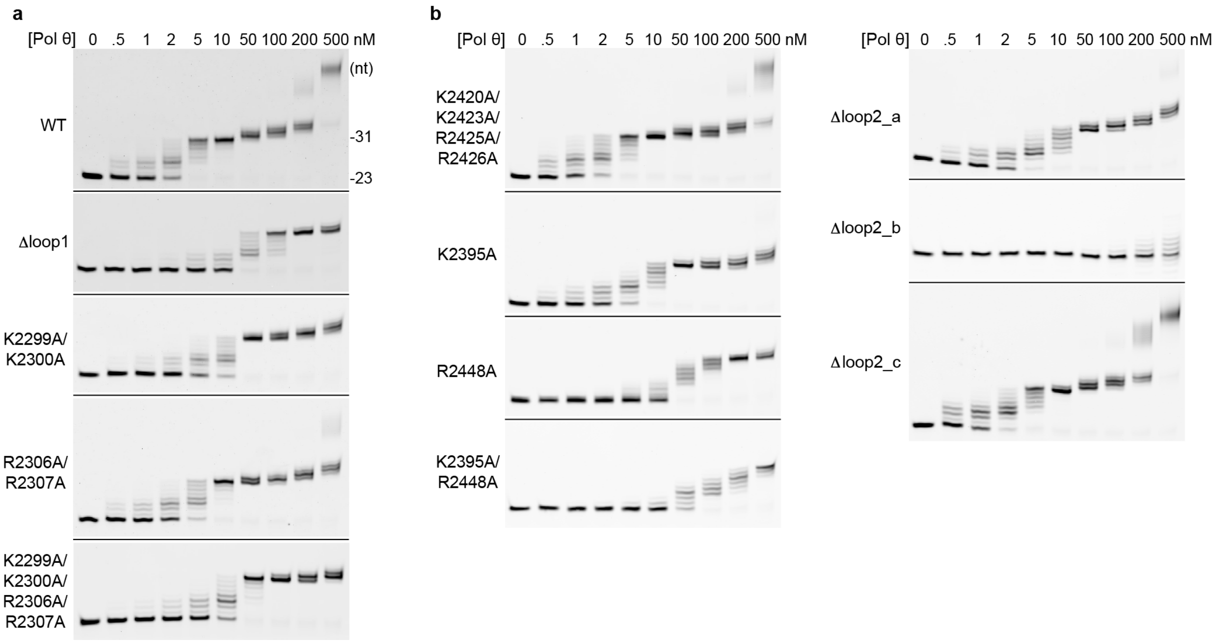

Fig. S9. Representative denaturing gels showing the DNA synthesis activity of WT and mutant LcPol θ-pol on the hairpin substrate HP [3,9]. **(a)** Gels for WT and loop 1 deletion and mutations. **(b)** Gels for loop 2 deletions and mutations. The control samples without enzymes are shown in the -E lanes.

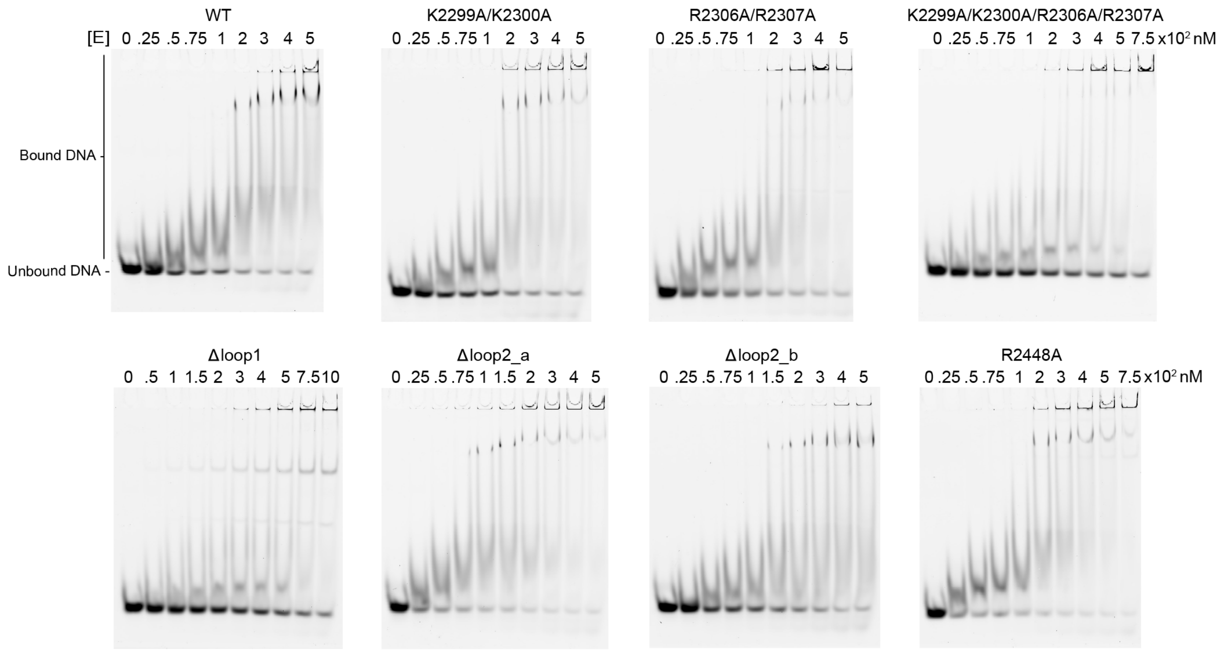

Fig. S10. Representative EMSA gels showing the binding of WT and mutant LcPol θ-pol to the hairpin substrate HP [3,9] at various protein concentrations.

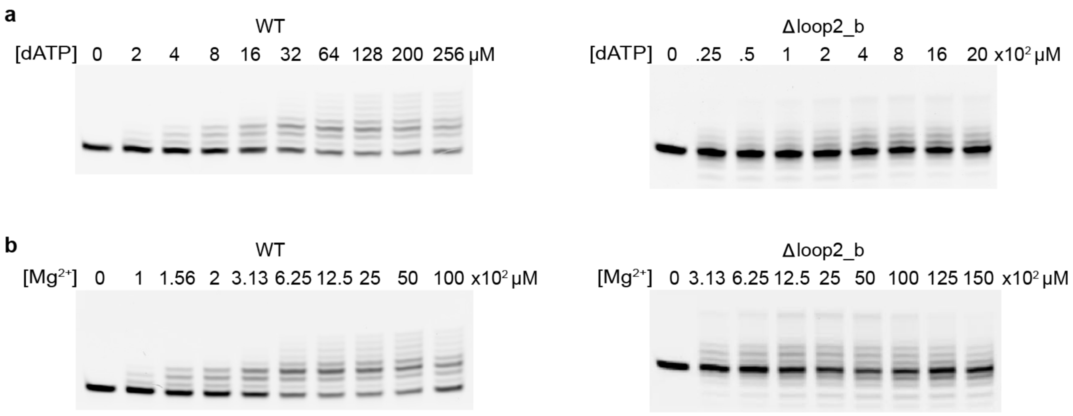

Fig. S11. Representative denaturing gels showing HP [3,9] extension by WT LcPol θ-pol and Δloop2_b under steady-state conditions with increasing concentrations of **(a)** dATP or **(b)** Mg^{2+}.

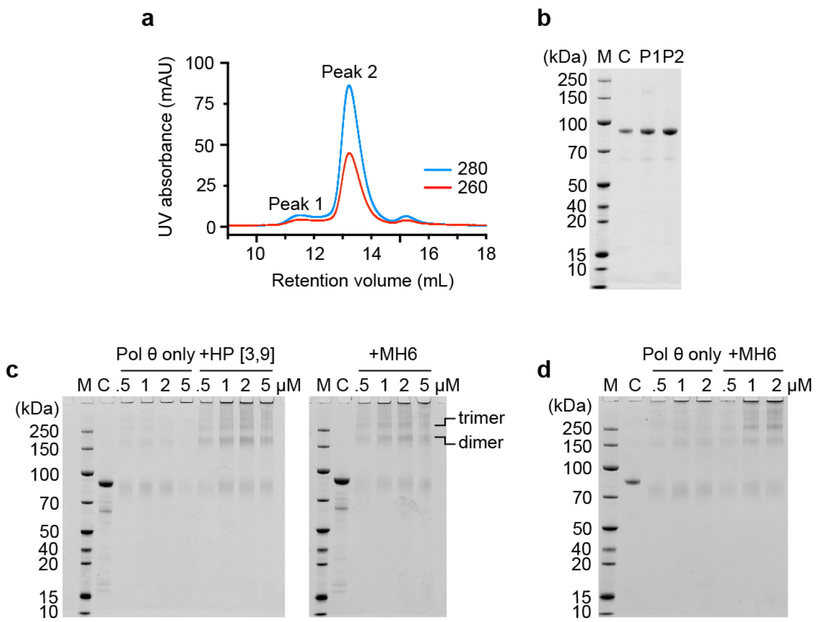

Fig. S12. Oligomerization of LcPol θ-pol. **(a)** Gel-filtration profile of LcPol θ-pol (84 kDa). **(b)** SDS-PAGE confirming the composition of peaks from gel-filtration. C, LcPol θ-pol before gel-filtration; P1, peak 1; P2, peak 2. **(c, d)** SDS-PAGE indicating the formation of LcPol θ-pol **(c)** and Δloop2_c **(d)** oligomers after crosslinking in the presence of DNA. C, negative control without glutaraldehyde treatment. The positions of a dimer and a trimer are marked on the side.

Table S1. Cryo-EM data collection, refinement, and validation statistics

	Complex Ia	Complex Ib	Complex II
PDB Code	8EF9	8EFC	8EFK
EMDB Code	EMD-28075	EMD-28078	EMD-28084
**Data collection and processing**			
Magnification	81kx		105kx
Voltage (kV)	300		300
Electron exposure ($e^-/Å^2$)	72		67
Defocus range (μm)	-0.8 to -1.6		-1.0 to -2.0
Pixel size (Å)	1.06		0.826
No. of micrographs			
Collected	6,409		4,357
Used	6,311		4,208
Symmetry imposed	C1		C1
No. particles used	825,204	406,678	288,920
Map resolution (Å)	2.4	2.8	3.0
Corrected, FSC=0.143			
**Refinement**			
Cross-correlation			
CC_mask	0.73	0.67	0.82
CC_volumes	0.71	0.66	0.64
CC_peaks	0.64	0.61	0.62
Model composition			
Protein	4715	4715	4715
DNA	493	755	273
Ligands/Mg^{2+}	32	32	31
B-factors			
Protein	17.8	10.8	28.8
DNA	35.0	28.4	73.5
Ligands/Mg^{2+}	15.3	4.9	42.7
R.m.s deviations			
Bond lengths (Å)	0.007	0.005	0.008
Bond angles (º)	0.752	0.734	0.731
Ramachandran			
Favored (%)	94.0	94.7	95.9
Allowed (%)	5.8	5.0	3.9
Outlier (%)	0.2	0.3	0.2
Validation			
MolProbity score	1.9	2.0	1.5
Clashscore	9.4	12.4	4.9

Table S2. DNA sequences used in the present work

Oligonucleotide	Sequence
19/29 mer_primer	5′TGCTGTGAGGCATCCGTAG
19/29 mer_template	5′GCAGTCAGCTCTACGGATGCCTCACAGCA
MH6_primer	5′TTTTTTTTTTGCCCGC
MH6_template	5′TTTTTTTTTTGCGGGC
HP [2,3]	5′TTTTTTTTGGTTTCC
HP [3,3]	5′TTTTTTTTGGCTTTGCC
HP [4,3]	5′TTTTTTTTGGCGTTTCGCC
HP [5,3]	5′TTTTTTTTGGCGCTTTGCGCC
HP [6,3]	5′TTTTTTTTGGACGCTTTGCGTCC
HP [3,6]	5′TTTTTTTTGGCTTTTTTGCC
HP [3,9]	5′TTTTTTTTGGCTTTTTTTTTGCC
HP [3,12]	5′TTTTTTTTGGCTTTTTTTTTTTTGCC
HP [3,15]	5′TTTTTTTTGGCTTTTTTTTTTTTTTTGCC
